# Improving CT Sinus Reporting for Endoscopic Sinus Surgery Using the CLOSE Criteria: A Quality Improvement Project

**DOI:** 10.7759/cureus.96762

**Published:** 2025-11-13

**Authors:** Teck Yon Lee, Hamish Iyer, Ashutosh Ranade, Richard Steven, Sophia Wong Ching Hwai

**Affiliations:** 1 General Surgery, NHS Fife, Kirkcaldy, GBR; 2 Geriatrics, Victoria Hospital, Kirkcaldy, GBR; 3 Radiology, Victoria Hospital, Kirkcaldy, GBR; 4 Otolaryngology - Head and Neck Surgery, Victoria Hospital, Kirkcaldy, GBR; 5 Respiratory Medicine, Northern Care Alliance NHS Foundation Trust, Greater Manchester, GBR

**Keywords:** ct sinus, functional endoscopic sinus surgery (fess), neuroradiology, quality improvement projects, radiology template, sinonasal anatomical variants, sinus disease, structured reporting template

## Abstract

Introduction

Pre-operative CT sinus reports are essential for identifying high-risk anatomical variants during functional endoscopic sinus surgery (FESS). The widely accepted CLOSE criteria provide a structured framework for this reporting. The objective of this quality improvement (QI) project was to assess the completeness of CT sinus reporting for patients likely to undergo FESS using the CLOSE framework and introduce interventions to standardise reporting and improve patient safety.

Methods

This three-cycle QI project was conducted at a single NHS health board, reviewing 92 retrospective CT sinus reports between February 2025 and September 2025. Educational interventions were implemented following each cycle, including the distribution of a poster to relevant radiology departments and an NHS-partnered teleradiology company, the collection of feedback, and the reinforcement of changes at departmental meetings. An accompanying survey was sent out to ENT consultants to gather qualitative data. Compliance rates between cycle one and cycle three were compared using the chi-square test.

Results

Comparing cycle one to cycle three, a significant increase in compliance was observed. Full documentation of all CLOSE components rose from a baseline of 8/51 (15.7%) to 11/21 (52.4%). For reports demonstrating high-risk anatomical variants relevant to surgical planning, overall compliance rose from 5/51 (9.8%) to 9/21 (42.9%) (χ^2^=10.375, p<0.01).

Our predominantly Scottish cohort showed that Keros type II and sellar sphenoid sinus pneumatisation were the most common findings, with 36/92 (39%) patients exhibiting supraorbital pneumatisation.

The consultant survey (n=11) showed that identifying high-risk anatomy was the most important feature (11/11 (100%) respondents). While 9/11 (82%) respondents were familiar with the CLOSE framework, 5/11 (45%) considered the existing CLOSE criteria only partly sufficient.

Conclusion

The successful implementation of a systematic, recognised checklist like the CLOSE criteria substantially improved the quality and consistency of CT sinus reporting. This replicable methodology enhances surgical planning, improves patient safety, and strengthens communication between radiology and ENT departments.

## Introduction

Functional endoscopic sinus surgery (FESS) is a common procedure that restores sinus drainage and airflow in conditions such as chronic rhinosinusitis, nasal polyps, tumours, and complications of acute rhinosinusitis. FESS can alleviate symptoms of nasal congestion, impaired breathing, hyposmia, and facial pain, improving the quality of life for individuals affected. Through small incisions of the osteomeatal complex, the surgeon can gain access to diseased mucosa and bone obstructing the sinuses, re-establishing mucociliary clearance and patency of drainage pathways [1,2].

Pre-FESS CT scanning is used to identify potential variations and high-risk anatomical structures. The resulting CT sinus reports must convey all necessary pre-operative information to enable the surgeon to prepare and anticipate anatomical variations that could predispose patients to surgical complications. Inconsistent or variable reporting of these variants can lead to unrecognised high-risk anatomy, increasing the likelihood of intraoperative complications such as cerebrospinal fluid (CSF) leaks, orbital injury, or haemorrhage from the anterior ethmoidal or internal carotid arteries [1]. The commonly used CLOSE criteria incorporate the following critical variants: Cribriform plate, Lamina papyracea, Onodi cell, Sphenoid sinus pneumatisation, and (anterior) Ethmoidal artery [3,4]. The CLOSE criteria provide a simple means of recalling that structures need reporting, making it easier for radiologists to report and ENT surgeons to identify high-risk injury sites before surgery [1].

By implementing a standardised and structured template grounded in evidence-based guidelines, we can ensure that every patient undergoing sinus surgery is appropriately screened for these variants, reducing the risk of surgical complications [5].

This quality improvement (QI) project aimed to assess the completeness of CT sinus reporting for patients likely to undergo FESS using the CLOSE framework, and to implement interventions to standardise reporting and improve patient safety.

## Materials and methods

Study design and setting

This QI project was conducted at Victoria Hospital, Kirkcaldy, and Queen Margaret Hospital, Dunfermline, between February 2025 and September 2025. A total of three Plan-Do-Study-Act cycles were completed. The study was designed and reported in accordance with the SQUIRE 2.0 guidelines for reporting QI studies [6].

Ethical approval was not required, as this was a service evaluation project with the aim of improving radiology reporting standards. All data were anonymised and collected retrospectively. The project was registered with the Fife Clinical Effectiveness Team (Record ID 41663).

The project aligns with the principles set forth by the Royal College of Radiologists, which emphasises that radiological reports should be accurate, complete, and directly address the clinical query [7]. Our primary goal was to standardise reporting for patients undergoing FESS by introducing a structured template that ensures key surgical landmarks are consistently documented. This intervention, based on the CLOSE criteria, was designed to reduce variation in reporting, enhance communication between radiology and ENT departments, and ultimately improve patient safety by ensuring consistent documentation of key surgical landmarks.

Data collection: cycle one

Between February 2025 and May 2025, we retrospectively reviewed 51 CT sinus reports requested for patients with sinonasal disease likely to undergo FESS. Each report was assessed against the CLOSE criteria, with data collected under the following headings: (1) Clinical indication, whether the request or report indicated that the patient was being worked up for potential FESS; (2) Anatomical variants, identification of any relevant high-risk anatomical variants corresponding to the CLOSE criteria; (3) Reporting completeness, whether each anatomical variant was not documented, partially documented, or fully documented in the radiology report; (4) Overall surgical relevance, for each case, we reported whether the patient had high risk anatomy that was not explicitly documented in the report, posing a significant risk to patients likely to undergo FESS.

CT reports were selected using a random sampling method between February and May, while all available CT sinus reports were reviewed between August and September to increase the overall sample size. All data were collected retrospectively and fully anonymised before analysis.

This first cycle, therefore, allowed us to assess both the prevalence of surgically relevant high-risk variants in our population and the level of compliance of routine CT sinus reporting with the CLOSE framework.

Intervention

Following completion of the first data collection cycle, an educational intervention was developed to improve the consistency of CT sinus reporting.

We designed an educational poster summarising the CLOSE criteria and highlighting its clinical importance for patients likely to undergo FESS. The poster was distributed via email and displayed in relevant radiology reporting areas and affiliated departments.

Informal feedback was sought from radiologists to refine the design of the poster and ensure the content was clear, concise, and practical for day-to-day reporting. Minor modifications were incorporated in response to this feedback before wider circulation.

ENT consultant survey

To supplement radiology data, we designed a short online questionnaire aimed at ENT consultants at Victoria Hospital, Kirkcaldy, and Ninewells Hospital, Dundee. The survey consisted of five questions: (1) Do current radiology CT sinus reports provide you with sufficient information for FESS planning? (2) In your opinion, what are the most important features of a CT sinus report for patients likely to undergo endoscopic sinus surgery? (3) Are you familiar with the CLOSE criteria for CT sinus reporting? Do you find it sufficient for surgical planning? (4) Which of the following anatomical features should be clearly addressed in every CT sinus report for surgical planning? (5) What aspects of the report delivery do you find most important? 

The survey was distributed by email in July 2025, and we received 11 responses. This qualitative dataset was analysed descriptively.

Data collection: cycle two

Between July 2025 and August 2025, we repeated the audit cycle by reviewing 20 new CT sinus reports requested for patients likely to undergo FESS. Data were collected using the same methodology as in cycle one.

Intervention

A formal departmental radiology meeting was held in September 2025, during which the project findings were presented. This included a summary of the baseline data, ENT survey results, and the impact of the poster intervention. The meeting provided an opportunity to re-emphasise the importance of documenting CLOSE criteria and to encourage discussion on sustainable implementation.

To maximise reach, the educational poster was distributed more widely, including circulation to Medica Reporting Limited (commonly referred to as Medica), an NHS-partnered teleradiology company that reports a portion of local CT scans, and posting on the departmental channel.

Data collection: cycle three

A third cycle was performed in September 2025, again assessing 21 new CT sinus reports using the same methodology as in cycle one.

Data analysis

All data were entered into a secure spreadsheet and analysed descriptively. Categorical data were presented as frequencies and percentages. Where appropriate, comparative proportions between cycle one and cycle three were analysed using the chi-square test. Survey responses were presented as summary statistics and simple bar charts.

## Results

A total of 92 CT sinus scans were reviewed across three audit cycles between February 2025 and September 2025. Table 1 summarises the proportion of CT sinus reports documenting each component of the CLOSE criteria: Cribriform plate (C), Lamina papyracea (L), Onodi cell (O), Sphenoid sinus (S), and Ethmoidal artery (E), graded as not documented, partially documented, or fully documented.

**Table 1:** Proportion of CT sinus reports documenting each component of the CLOSE criteria across all cycles n, sample size.

CLOSE Component	Documentation Status	Cycle 1 (n=51)	Cycle 2 (n=20)	Cycle 3 (n=21)
C (Cribriform plate)	Fully documented	20 (39.22%)	9 (45.00%)	13 (61.90%)
	Partially documented	6 (11.76%)	3 (15.00%)	1 (4.76%)
	Not documented	25 (49.02%)	8 (40.00%)	7 (33.33%)
L (Lamina papyracea)	Fully documented	29 (56.86%)	9 (45.00%)	15 (71.43%)
	Partially documented	1 (1.96%)	5 (25.00%)	1 (4.76%)
	Not documented	21 (41.18%)	6 (30.00%)	5 (23.81%)
O (Onodi cell)	Fully documented	26 (50.98%)	8 (40.00%)	13 (61.90%)
	Partially documented	2 (3.92%)	4 (20.00%)	0 (0.00%)
	Not documented	23 (45.10%)	8 (40.00%)	8 (38.10%)
S (Sphenoid sinus)	Fully documented	12 (23.53%)	5 (25.00%)	5 (23.81%)
	Partially documented	0 (0.00%)	6 (30.00%)	8 (38.10%)
	Not documented	39 (76.47%)	9 (45.00%)	8 (38.10%)
E (Ethmoidal artery)	Fully documented	26 (50.98%)	9 (45.00%)	11 (52.38%)
	Partially documented	1 (1.96%)	2 (10.00%)	3 (14.29%)
	Not documented	24 (47.06%)	9 (45.00%)	7 (33.33%)

**Figure 1:**
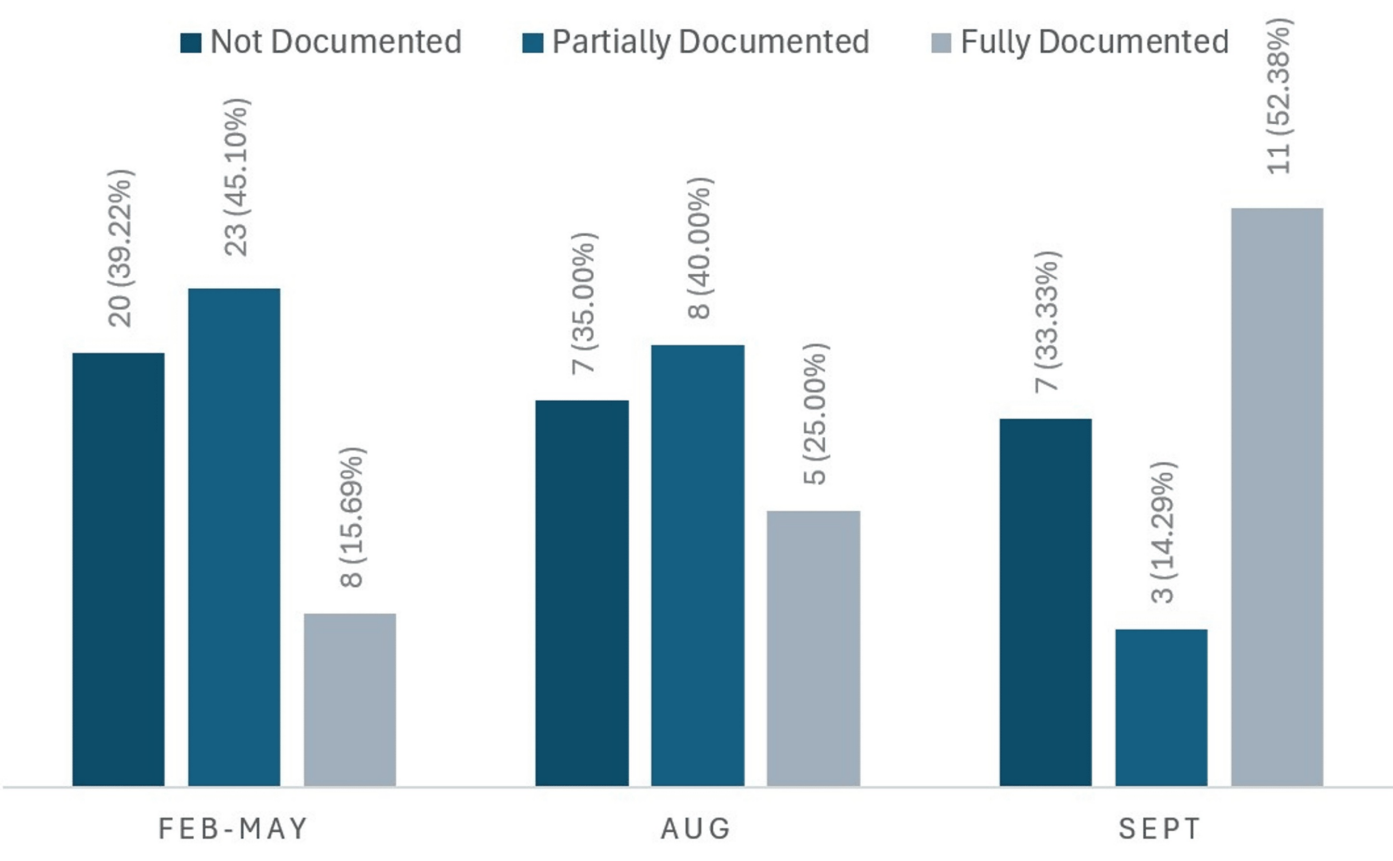
Bar chart demonstrating overall CLOSE compliance across three audit cycles

Figure 1 demonstrates the progressive improvement in overall CLOSE compliance between the three cycles. Following the introduction of the educational poster and departmental meeting presentation, full documentation compliance rose significantly, increasing from a baseline of 8/51 (15.7%) to 11/21 (52.4%) in cycle three. 

**Figure 2:**
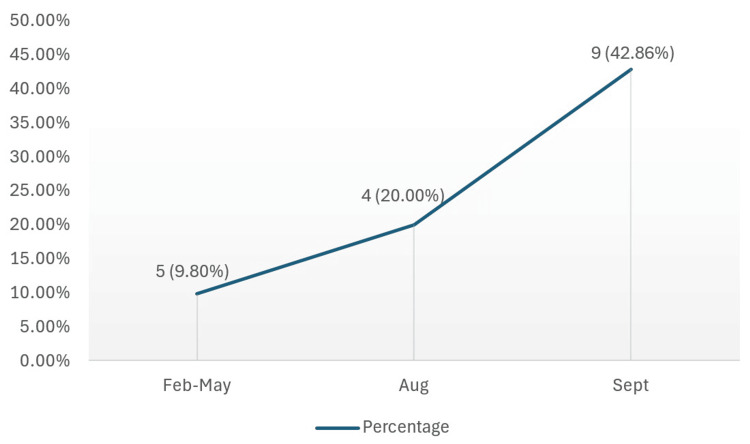
Percentage of CT sinus scans that are fully CLOSE-compliant, contain high-risk anatomy, and are clinically relevant for FESS FESS, functional endoscopic sinus surgery.

Figure 2 summarises the proportion of reports that were fully compliant with the CLOSE criteria, demonstrated high-risk anatomical variants, and were clinically relevant for FESS. Comparing cycle one and cycle three, a chi-square test was performed, which showed a statistically significant improvement in overall compliance following intervention from 5/51 (9.8%) to 9/21 (42.9%) (χ2=10.375, p < 0.01).

Table 2 presents the prevalence of individual sinonasal anatomical variants identified within our cohort, which were predominantly Scottish patients. The most common findings were Keros type II and sellar sphenoid sinus pneumatisation. Notably, supraorbital pneumatisation was identified in over one-third of patients, a finding which increases the risk of damage to the anterior ethmoidal arteries during surgery.

**Table 2:** Prevalence of sinonasal anatomical variations in the study population K1, Keros type I; K2, Keros type II; K3, Keros type III; n, sample size.

CLOSE component	Anatomical variant	Percentage (n=92)
C (Keros classification)	K1	16 (17.39%)
	K2	60 (65.22%)
	K3	16 (17.39%)
L (lamina papyracea dehiscence)	Lamina papyracea intact	88 (95.65%)
	Dehiscence present	4 (4.35%)
O (presence of Onodi cell)	Present	8 (8.70%)
	Absent	84 (91.30%)
S (sphenoid sinus pneumatisation)	Conchal	6 (6.52%)
	Pre-sellar	18 (19.57%)
	Sellar	53 (57.61%)
	Post-sellar	15 (16.30%)
E (presence of supraorbital pneumatisation)	Present (bilateral/unilateral)	36 (39.13%)
	Absent	56 (60.87%)

A total of 11 ENT consultants from Victoria Hospital, Kirkcaldy, and Ninewells Hospital, Dundee, participated in the questionnaire, achieving a response rate of over 11/18 (60%).

Question one

One (9%) respondent reported that current CT sinus reports consistently provide sufficient information for FESS planning; however, 7/11 (64%) respondents reported that it is frequently sufficient.

Question two

11/11 (100%) respondents agreed that identification of high-risk anatomy was the single most important feature of a CT sinus report; this was followed by description of bone erosion or thinning (9/11 (82%)) and clear description of key surgical landmarks (5/11 (46%)).

Question three

9/11 (82%) respondents were familiar with the CLOSE criteria, although 5/11 (45%) considered it only partly sufficient for comprehensive pre-operative planning, indicating potential areas for refinement or local adaptation.

Question four

The five most frequently named anatomical structures considered essential for reporting were the cribriform plate, lamina papyracea, Onodi cell, anterior ethmoidal artery, the optic nerve, and the internal carotid artery course or dehiscence.

Question five

The majority of respondents (7/11 (64%)) valued inclusion of additional relevant findings such as skull base and orbital and dental pathology. Over half (6/11 (55%)) preferred a concise summary at the end.

## Discussion

This QI project demonstrated that implementation of an educational intervention based on the CLOSE criteria substantially improved the quality and consistency of CT sinus reporting within our health board. Across three audit cycles, full documentation of all CLOSE components relevant for surgical planning increased from 5/51 (9.80%) to 9/21 (42.86%) (p < 0.01), representing a statistically significant improvement. These findings support the use of structured reporting frameworks to enhance communication between radiologists and ENT surgeons, particularly for patients undergoing FESS.

Our findings are consistent with prior studies showing that anatomical variants that increase surgical risk are common but often underreported. A survey of Australian otolaryngologists indicated that most responders (94.6%) felt that formal CT-sinus reporting offered little extra clinical information compared with their own assessment of the images. They specifically requested more information regarding high-risk surgical areas (57%) and anatomical variations (28%) [3].

Another study showed that before implementation, the rate of correctly identified anatomical variants was only 24%; this rose significantly to 84% after implementation of the CLOSE checklist for ENT trainees [8].

The result of this project has several important clinical implications that go beyond simply improving completeness. Structured reporting ensures that key anatomical variants such as deep olfactory fossa (Keros type III), lamina papyracea dehiscence, optic nerve, and carotid canal dehiscence are identified and described. This allows surgeons to anticipate difficult anatomy preoperatively, reducing complications such as CSF leaks and orbital and neurovascular injury [9]. While these adverse events are infrequent, their consequences are often devastating [10]. Furthermore, missing critical anatomy in reports may expose institutions and clinicians to medicolegal risk if complications arise.

Beyond optimising patient safety, standardised reporting also contributes to sustainability by reducing the need for repeat CT scans. When a report is incomplete or lacks critical surgical detail, surgeons may request a second, dedicated, or contrast-enhanced CT, adding unnecessary radiation dose, cost, and delay. More broadly, the heavy reliance on complex imaging carries an environmental cost. The energy-intensive operation of medical imaging equipment, particularly CT scanners, contributes significantly to the carbon footprint of healthcare [11].

Limitations

This QI project has several limitations that should be acknowledged. Firstly, the study was conducted within a single NHS health board (NHS Fife), involving a relatively small number of CT sinus reports over a limited period. Although sufficient for demonstrating local improvement, the sample may not be representative of wider UK radiology practice. Multicentre collaboration would be required to build on these findings. To ensure the sustainability of these improvements, the structured CLOSE reporting template must be formally adopted by NHS radiology departments. Furthermore, ongoing education for new staff and scheduled repeat audits will allow for stronger, long-term adherence to this structured template. 

The CLOSE criteria, while widely used, still allow for subjective interpretation, particularly for reports that are partially documented. Reports that provide only a general statement, 'no significant anatomical variants found', without explicitly detailing key surgical areas, may be inadequate for guiding ENT surgeons and carry the risk of failing to document a critical anatomical variant.

It is also noted from our survey results that 5/11 (45%) ENT surgeons described the CLOSE criteria as only partially sufficient. This is due to the highly diverse nature of sinonasal anatomy, which varies across populations. Consequently, uncommon anatomical variants, some of which carry significant surgical risk, may be underreported when relying solely on the existing criteria. Future research should focus on expanding the CLOSE checklist to incorporate a wider spectrum of clinically relevant anatomical variants.

Lastly, although improved documentation of high-risk anatomy is expected to reduce intraoperative complications, this project did not measure surgical outcomes or complication rates before and after intervention. Future studies could explore whether enhanced structured reporting directly correlates with safer FESS procedures and improved clinical outcomes.

## Conclusions

This project successfully demonstrated that the application of a widely recognised and systematic checklist directly correlates with improved adherence to reporting standards and enhanced patient safety. Following the integration of the CLOSE criteria, our department achieved a substantial improvement in report completeness. This project’s methodology is replicable in other radiology centres that report CT sinus scans, providing a framework to enhance surgical planning, improve patient safety and strengthen communication between radiology and ENT.
